# Some Considerations about Winter Colony Losses in Italy According to the Coloss Questionnaire

**DOI:** 10.3390/insects13111059

**Published:** 2022-11-16

**Authors:** Franco Mutinelli, Anna Pinto, Luciana Barzon, Marica Toson

**Affiliations:** 1 NRL for Honey Bee Health, Istituto Zooprofilattico Sperimentale delle Venezie, 35020 Legnaro, PD, Italy; 2 Communication Laboratory, Istituto Zooprofilattico Sperimentale delle Venezie, 35020 Legnaro, PD, Italy; 3 Epidemiology Laboratory, Istituto Zooprofilattico Sperimentale delle Venezie, 35020 Legnaro, PD, Italy

**Keywords:** winter colony losses, Coloss questionnaire, honeybee, treatment, *Varroa destructor*

## Abstract

**Simple Summary:**

The Italian beekeeping industry has grown steadily during the last decade, according to data from the national beekeeping registry. Nevertheless, winter colony losses remain a matter of concern for beekeepers in Italy, and administration of the questionnaire defined by the Coloss Association could contribute to a better understanding of this phenomenon. Since 2008, data on winter colony losses have been gathered through the Coloss questionnaire at European Union, European, and extra-European levels. In Italy, these data have been collected since 2008 in hard-copy form and online since 2019–2020. However, since the responding beekeepers did not represent all Italian regions over the years, the whole questionnaire administration period (2008–2021) could not be reasonably considered for analysis. Hence, we included only the periods 2014–2015 and 2020–2021. Among the possible causes of colony losses were queen-related problems, natural disasters, and dead or empty colonies, since these questions remained unchanged over time. We also took account of responses related to treatments against *Varroa* mite infestation.

**Abstract:**

The Italian beekeeping industry has grown steadily during the last decade, according to data from the national beekeeping registry, which came into existence in February 2015. Winter colony losses remain a matter of concern for beekeepers in Italy, and administration of the questionnaire defined by the Coloss Association could contribute to a better understanding of this phenomenon. To evaluate the percentage trends over time in honeybee colony losses arising from various causes, we used the quasi-binomial generalized linear modelling (GzLM) approach, taking the year as an independent variable. We set our level of significance at 5% and performed the data analysis only for the seven regions that sent data continuously from 2014 to 2020. We considered the percentage of losses due to queen-related problems, natural disasters, and dead or empty colonies, given that these questions remained unchanged over the years. The survey also revealed that the percentage trend for respondents using drone brood removal showed a significant increase. In general, the percentage of colony losses due to queen-related problems remained lower than 8%, and the percentage of colony losses associated with natural disasters was very low (<2%). The mean percentages of losses due to dead or empty colonies ranged from 6 to 17% in the considered period. In addition, we took account of the responses relating to treatments against *Varroa* mite infestation, given the importance attributed to this honeybee parasite. Unlike the other variables, we calculated the percentages related to the types of beekeeper treatments against *Varroa destructor* based on the respondents, not on the colonies. What emerged was that almost every beekeeper used at least one type of treatment against *V. destructor*. In general, the trend of respondents appeared stable at 0.3% during the last four years.

## 1. Introduction

The Italian beekeeping industry has grown steadily during the last decade, following the implementation of the national beekeeping registry, in accordance with the Ministry of Health Decrees of 4 December 2009 and 11 August 2014, which became fully operative in 2015 [1,2]. The Interdirectorial Decree (Ministries of Health and Agriculture) of 27 November 2017 further integrated the national beekeeping registry [3]. This system was actually developed in advance of, and is now in agreement with, Regulations 2019/2035 and 2022/1345 defining the rules governing establishments keeping terrestrial animals and with more recent Italian legislation [4,5,6,7]. The figures for the Italian national beekeeping registry as of 30 June 2022 are as follows: 71,104 beekeepers, 175,281 apiaries, 1,767,390 colonies, and 278,982 nuclei (referred to as swarms in the beekeeping registry). The average densities are 0.58 apiaries/km^2^ and 0.23 beekeepers/km^2^, with peaks of 1.6 apiaries/km^2^ and 0.92 beekeepers/km^2^ in the north. Most Central–Northern regions and some areas of Southern Italy seem the most suited to apiculture (https://www.vetinfo.it/j6_statistiche/#/report-pbi/45) (accessed on 4 October 2022).

The health status of honeybee colonies has been investigated across the Italian territory [8,9,10,11], focusing on nature reserves [12,13] and at European Union level [14,15], with a view to better understanding the phenomenon of colony losses and the possible risk factors involved. Spring honeybee losses were also investigated in Italy in spring 2008 in relation to the use of neonicotinoid-coated corn seeds [16,17,18]. Winter losses have been studied in many other countries in Europe [19,20,21,22,23,24,25,26,27,28,29,30,31,32,33,34,35,36,37,38,39,40] and beyond [41,42,43,44,45,46,47,48]. The possible impacts on colony losses of Varroa mite infestation and its control [49,50,51], as well as of weather conditions [52,53,54,55,56], have also been investigated.

Winter colony losses are a cause for concern for the beekeeping industry of Italy, and the first reports on this topic go back to 2007/2008 [57,58]. Honeybee colony losses recorded in Italy in the winter of 2007–2008 were 30–40% in the Northern part of the country and 10–30% in central and southern areas, based on oral reports from beekeepers’ associations. Data from anonymous questionnaires distributed and collected during beekeepers’ meetings confirmed these oral reports [58]. The limited availability of data on losses prevents a better understanding of the phenomenon and of causative factors. It does appear, however, that the reported losses are attributable mainly to inadequate and/or improper control of *Varroa destructor* infestation and interactions between the mite and other pathogens and only partially to inadequate apicultural techniques. The questionnaires used were processed locally and developed in the framework of COST Action FA0803 “COLOSS” (Prevention of honeybee COlony LOSSes). The latter was then formalized as the Coloss questionnaire, which constitutes an output of the Coloss Association (www.coloss.org) (accessed on 4 October 2022) [58]. Further improvement in the questionnaire’s administration and targeting of different specific topics each year could contribute to a better understanding of this phenomenon. Data on winter losses have been collected through the Coloss questionnaire at European Union, European, and extra-Europe levels since 2008 [59,60,61,62,63,64]. In Italy, these data have been gathered since 2008 (in hard-copy format and online since 2019–2020). However, since the responding beekeepers did not represent all Italian regions over the years, we could not reasonably consider the whole questionnaire administration period (2008–2021) for analysis. Hence, our analysis was limited to the periods 2014–2015 and 2020–2021.

The goal of our paper was to present the data collected through the Coloss questionnaire in Italy, quantify colony losses, and consider the possible causes. Among the collected data, we devoted attention to those related to treatments carried out by beekeepers against the parasitic mite *V. destructor*. Lastly, we considered critical issues encountered in conducting the Coloss survey in Italy, particularly in relation to the very limited participation recorded since the beginning of the monitoring project.

## 2. Materials and Methods

The Coloss questionnaire has been used in Italy since 2008 [58,59,60,61,62,63,64] to collect data on winter honeybee colony losses. It consists of about 27 questions which mainly require numerical entries and are closed-ended (single or multiple choice), except for a few open-ended questions. As the questionnaire has varied slightly from year to year, the number of questions has also varied, from a minimum of 21 to a maximum of 28. Of these, about 15 were mandatory for continuation, while the remainder were optional. Only the questions listed in Table S1 were included in the present study. The others were deemed irrelevant to the aim of this study. A copy of the full questionnaire is available as Table S2. 

Regarding questionnaire administration and data collection, until 2018–2019, the survey was disseminated via the website www.izsvenezie.it (accessed on 4 October 2022). Here, it was possible to download the questionnaire, fill it in on paper, and then email it for data collection purposes. The questionnaire was further advertised via an official note from the Ministry of Health emailed to national and local beekeepers’ associations and beekeeping magazines and distributed during beekeeping meetings. In 2019–2020, online entry was introduced. The questionnaire, which was computerized using the Lime Survey web application, could therefore be filled in directly online or downloaded and filled in on paper as in previous years. Data collection took place between the end of March and mid-June each year. An introductory text was placed both at the beginning of the hard copy of the questionnaire and online at the website www.izsvenezie.it (accessed on 4 October 2022). All participants were therefore fully informed about the research and its purpose. 

No sensitive data were collected through the questionnaire; the only personal information requested was the post/zip code and name of the city/town/village near to the respondent’s apiary. Accordingly, the research did not require review or approval by the Ethics Committee. Nevertheless, we adopted specific participant protection procedures to comply with privacy policy. Since 2019–2020, participants in the online survey have been requested to consent to a privacy agreement through a checkbox at the beginning of the online questionnaire. Before 2018–2019, i.e., when only the hard-copy version of the questionnaire was available, response to the questionnaire was deemed to constitute consent, considering that participants were invited to take part in the survey on a voluntary basis. The data were registered anonymously and handled in accordance with the EU General Data Protection Regulation [65].

To verify the presence of any monotonic increasing or decreasing trends in the percentages by year for honeybee colony losses due to various causes, we used the quasi-binomial generalized linear modelling (GzLM) approach [51], taking the year as an independent variable. We used R version 3.2. (©2015) software. We set our level of significance at 5% and carried out the data analysis only for the seven regions that sent data continuously from 2014 to 2020. We considered the percentages of colony losses due to queen-related problems, natural disasters, and dead/empty colonies, since these questions remained unchanged over the years. In addition, we also analyzed responses related to treatments against *Varroa* mite infestation. Concerning *V. destructor* control methods, for each treatment, we calculated the percentage of apiaries that underwent treatments in the year in question, determining the trend per year only for treatments with the highest percentages. 

## 3. Results

Table 1 presents the distributions of apiaries by region and year and of respondents with their colonies, as well as the percentages of respondents compared to the numbers of apiaries extracted from the Italian national beekeeping registry, according to the beekeepers’ answers to the Coloss questionnaire.

With the sole exception of Lombardia, Veneto, and Trentino Alto Adige, the number of respondents was quite low. The distribution of apiaries per region and year is based on data from the Italian national beekeeping registry (https://www.vetinfo.it/j6_statistiche/#/report-list/26) (accessed on 4 October 2022). Percentages >1 are highlighted in bold, and, in general, all percentages were very low, considering that the average density was 0.58 apiaries/km^2^, with peaks of 1.6 apiaries/km^2^ in the north of the country. In Friuli Venezia Giulia region (Northeast Italy), the percentage trend was found to be increasing, but appeared to be declining in the Veneto region. In general, the trend appears to have stabilized at 0.3% over the last four years (Table 1). The percentage numbers of respondents per apiary give an indication of respondent densities in the regions and in the country, based on registered establishments of managed honeybee colonies [4,5,6,7]. We considered the percentages of colony losses due to queen-related problems, natural disasters, and dead or empty colonies, since these questions remained unchanged over the years. The data are presented in Table S3.

**Figure 1:**
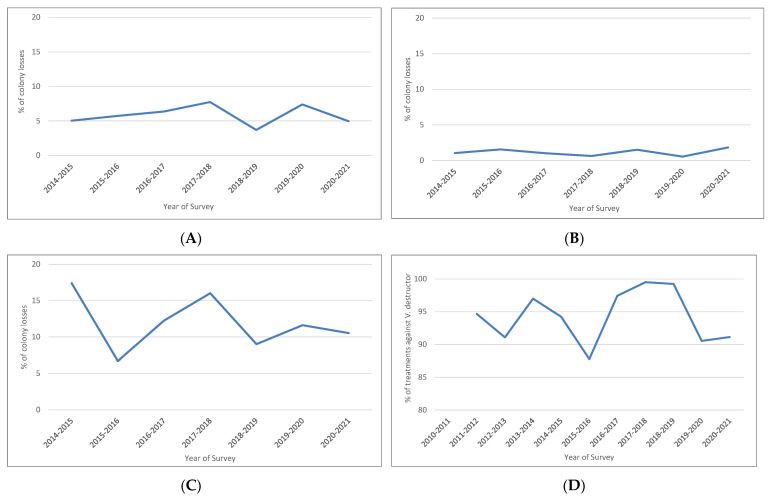
(**A**) Percentage of colony losses due to queen-related problems. (**B**) Percentage of colony losses due to natural disasters. (**C**) Percentage of losses arising from dead or empty colonies. (**D**) Percentage of treatments against *V. destructor* carried out by respondents.

Based on quasi-binomial generalized linear modelling (GzLM), the percentage trend for colony losses arising from queen-related problems appeared not to be significant (*p*-value = 0.628), as supported by the graphical analysis (Figure 1A). 

The trend for colony losses due to natural disasters was not significant (*p*-value = 0.297), as supported by the graphical analysis (Figure 1B). In the case of losses from dead or empty colonies (Figure 1C), the trend was not significant (*p*-value = 0.594). 

**Figure 2:**
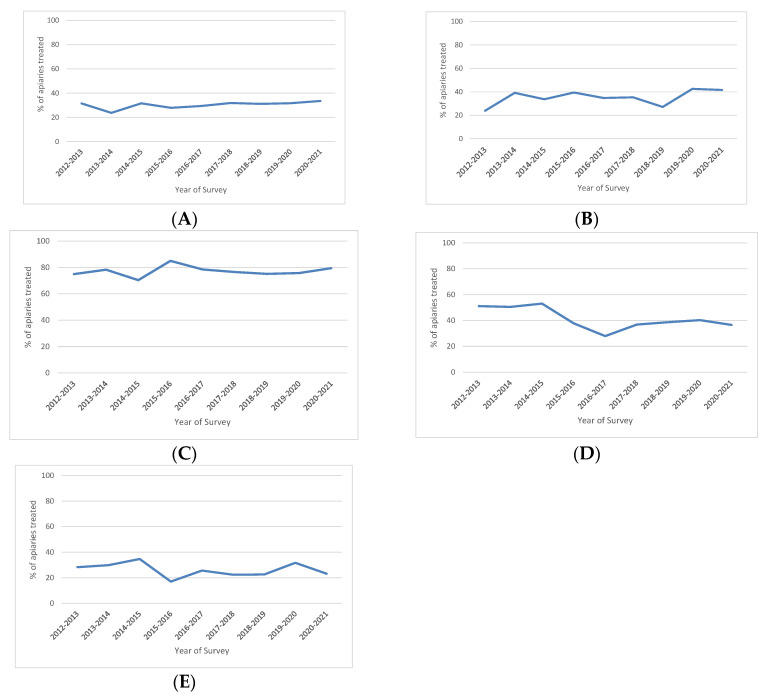
(**A**) Percentages of apiaries undergoing treatments based on drone brood removal. (**B**) Percentages of apiaries undergoing treatments using other biotechnical methods (e.g., trapping comb, complete brood removal, queen confinement). (**C**) Percentages of apiaries undergoing treatments with oxalic acid trickling. (**D**) Percentages of apiaries undergoing treatments with oxalic acid sublimation (evaporation). (**E**) Percentages of apiaries undergoing treatments with thymol (e.g., Apiguard, ApilifeVar).

Unlike the other previously considered variables, we based calculations of the percentages of types of beekeeper treatments against *V. destructor* on the respondents and not on the colonies. This revealed that almost every beekeeper used at least one type of treatment against *V. destructor* (Figure 1D), which is in agreement with the annual Italian national program for *V. destructor* control (www.izsvenezie.it) (accessed on 4 October 2022). Information about treatments was also available for previous years, but not for all the regions considered. For this reason, and in keeping with the other analyzed variables, we considered the years from 2010 to 2011 onwards. The trend was not significant (*p*-value = 0.922). For each treatment against *V. destructor*, we calculated the percentage of apiaries undergoing treatments that year, determining the trend per year only for treatments with the highest percentages (Figure 2A–E). 

Every year, the use of oxalic acid trickling stood in first place, with percentages of >70% and a peak of 85% in 2015–2016. In second place was oxalic acid sublimation (evaporation), which was mostly used in autumn–wintertime, with percentages ranging from 28% to 53%. There appeared to be a significant decrease (*p* < 0.01) in the trend for this method of applying oxalic acid. In the same regions, the trend in the use of drone brood removal (23–31%) was found to slightly significantly increase (*p* = 0.040). In the seven regions considered, the trends in the use of other biotechnical methods (23–42%) (e.g., trapping comb, complete brood removal, queen confinement) and thymol-based drugs (17–34%; e.g., Apiguard^®^, ApilifeVar^®^) were not significant. The high percentage in 2020–2021 of formic acid use (long-term; e.g., MAQS^®^) appeared unusual. We did not consider the periods 2010–2011 or 2011–2012 because of the incomplete data provided.

## 4. Discussion

The limited geographical participation in the Coloss questionnaire and the lack of continuity over the years placed several limitations on the relevance and reliability of the data collected, precluding any deeper analysis and substantial conclusions. Accordingly, we carried out data analysis only for the seven regions that sent data continuously from 2014 to 2020. Furthermore, we took account of the loss rates due to queen-related problems, natural disasters, and dead or empty colonies, since these questions remained unchanged over the years. In general, the percentage losses due to queen-related problems remained lower than 8%, which could be explained by the expertise of the beekeepers participating in the questionnaire. Furthermore, data collected in previous years through the Coloss questionnaire indicated queen-related losses of between 5 and 8.9% [59,60,61,62,63]. The percentage of lost colonies associated with natural disasters was very low (<2%) and could be attributed to the attention paid by beekeepers to the locations of their apiaries and to monitoring of weather-related natural events. Interestingly, the percentage losses arising from dead or empty colonies showed a decreasing trend. On average, this parameter ranged from 7 to 17% in the period considered, remaining around 15%, which is considered acceptable for Italian beekeepers. However, the acceptability range for winter colony losses differs among countries and regions and depends at least on operation size, management, geography, and climate [59], making comparisons difficult. If, for example, we consider the Coloss survey conducted in the winter of 2019–2020 [64] in countries recording overall winter loss rates similar to Italy (18.4%), the number of respondents was similar only for Ireland, being higher for Belgium and Slovakia and much higher for Germany. On the other hand, the number of colonies going into winter was much lower for Belgium and Ireland, higher for Slovakia, and extremely high for Germany. Furthermore, in the case of Italy, the collected data were not fully representative of the country, since geographical coverage was incomplete, based on the respondents’ origins. 

Almost every beekeeper reported performing at least one type of treatment against *V. destructor*, in line with the annual national control program [66]. Each year, the use of oxalic acid trickling stood in first place, ranging from >70% to a maximum of 85% in 2015–2016, followed by the use of oxalic acid sublimation (evaporation), ranging from 28 to 53% and used mostly in autumn–wintertime. As observed also in other European countries, oxalic acid-based drugs appeared among the veterinary medicines used most during both the active season after queen caging and in natural broodless periods [67]. Varroa mites severely affect honeybee health throughout the year, and their part in honeybee colony losses should be considered [49]. *V. destructor* control strategies should be developed and spread among beekeepers, and compliance with recommended treatment regimens should be consolidated to improve the survival of honeybee colonies over winter [50,51]. Varroa mite infestation control should definitely become part of routine honeybee colony management and best beekeeping practices to improve its efficacy and reduce colony losses [50,51,66].

The trends for treatments against *V. destructor,* as presented in Figure 2, demonstrate beekeepers’ attention to controlling this severe parasitic infestation. The use of drone brood removal exhibited a steady trend of between 20 and 40%, and other biotechnical techniques have likewise shown an increase in the last two years. This is in line with the national Varroa control program, beekeepers’ improved abilities to combine biotechnical tools with the application of veterinary medicinal products, and increased awareness about using less residue-producing drugs. This is related to the use of oxalic acid-based drugs administered by trickling in summertime and by evaporation mainly in autumn–wintertime. The use of thymol-based veterinary medicinal products was also reported to show a steady trend of between 20 and 30% during the last five years. Unexpectedly, in 2020–2021, the use of formic acid (long-term) reached an unusual high of 66%, considering that previously it had always remained below 10%. This could be partially explained by the fact that the characteristics of this veterinary medicinal product met beekeepers’ needs for an easier, safer application method for formic acid. An interesting topic to be considered in the framework of Varroa control strategies and surveys related to winter colony losses is the possible impact of weather on the winter mortality of honeybee colonies that has been recently investigated in Europe [50,52,53,54,55] and the USA [56].

All these interesting research topics could benefit from a tool like the Coloss questionnaire. It could be enriched each year with additional questions aimed at collecting information on specific fields, as has been done in the past for nutrition practices and queen replacement; in the future, questions could also potentially be included on weather conditions. 

Despite favourable conditions in terms of the availability and proper application of veterinary medicines against *V. destructor* [66], there has been growing interest in *Varroa*-resistant colonies in Italy, too. This has not been limited to research groups but has also involved beekeepers’ associations. In Sicily, interest was directed at *Apis mellifera sicula,* which apparently requires less or no treatment compared to other subspecies. Of course, this condition needs closer investigation, but the national *V. destructor* control program gives the option to define a specific protocol which can, in agreement with local veterinary services, differ from the national one [66], thus taking into account the features of this *Apis* subspecies and local geographic and climatic conditions [68].

Extensive contacts with beekeepers’ associations at national and local levels have been stimulated since the implementation of the Coloss questionnaire began. Furthermore, the Coloss questionnaire was issued on behalf of the Directorate General of Animal Health and Veterinary Medicines of the Italian Ministry of Health. Italian beekeepers’ participation in the questionnaire has always been poor due to little interest in the survey, lack of or limited confidence in surveys managed by government institutions, the concentration of potential respondents on their own business in springtime, or participation in association questionnaires. Even the availability of an online version of the questionnaire in both Italian and German has not helped increase the number of participants. Nevertheless, the geographical spread of the respondents has considerably increased in recent years, with greater involvement of Central–Southern Italy than in the past [63]. In 2020–2021, the number of participants was not exceptional, despite the presence of many more colonies (Table 1), which could be explained by the participation of more professional beekeepers than previously. According to Gray et al. [64], the estimated percentages of beekeepers in the 2019–2020 survey ranged from <1 to 17, with Italy scoring <1, confirming the results recorded every year since the first questionnaire in 2008. 

Given the role and relevance of beekeeping in Italy, we will continue to share the results of the Coloss questionnaire at national level through dedicated articles published in beekeeping journals and magazines, reports at beekeepers’ conferences and associative meetings, and by promoting the questionnaire through our website. In addition, we will stimulate beekeepers and their associations, as well as government and research institutions, to support participation in the Coloss questionnaire, despite the very limited interest shown to date. 

## Figures and Tables

**Table 1:** Distributions of apiaries by region and year and of respondents with their colonies, along with the percentages of respondents compared to the numbers of apiaries extracted from the Italian national beekeeping registry.

Region	2014–2015 *			2015–2016			2016–2017			2017–2018			2018–2019			2019–2020			2020–2021		
	N. of Apiaries *	N. of Respondents (N. of Colonies/Respondents)	% of Respondents/Apiaries	N. of Apiaries **	N. of Respondents (N. of Colonies/Respondents)	% of Respondents/Apiaries	N. of Apiaries **	N. of Respondents (N. of Colonies/Respondents)	% of Respondents/Apiaries	N. of Apiaries **	N. of Respondents (N. of Colonies/Respondents)	% of Respondents/Apiaries	N. of Apiaries **	N. of Respondents (N. of Colonies/Respondents)	% of Respondents/Apiaries	N. of Apiaries **	N. of Respondents (N. of Colonies/Respondents)	% of Respondents/Apiaries	N. of Apiaries **	N. of Respondents (N. of Colonies/Respondents)	% of Respondents/Apiaries
Emilia Romagna		2 (27)		8526	9 (145)	0.1	10,184	15 (1063)	0.1	11,467	11 (646)	0.1	12,740	4 (162)	0.0	14,598	9 (688)	0.1	15,711	18 (2971)	0.1
Friuli Venezia Giulia		22 (2443)		2686	4 (36)	0.1	3086	3 (1134)	0.1	3406	2 (22)	0.1	3689	12 (104)	0.3	4065	27 (400)	0.7	4332	48 (1099)	**1.1**
Lombardia		7 (128)		10,108	156 (2725)	**1.5**	12,752	150 (1867)	**1.2**	14,398	49 (1046)	0.3	15,306	7 (156)	0.0	17,303	13 (166)	0.1	19,087	77 (7957)	0.4
Piemonte		1 (10)		13,012	3 (50)	0.0	16,777	5 (193)	0.0	18,855	18 (664)	0.1	21,309	11 (479)	0.1	23,900	25 (648)	0.1	25,629	8 (1567)	0.0
Sicilia		1 (125)		4790	5 (195)	0.1	5986	3 (1423)	0.1	7105	3 (115)	0.0	8437	7 (2582)	0.1	10,398	8 (290)	0.1	11,447	44 (6327)	0.4
Trentino Alto Adige		9 (154)		5795	53 (1266)	0.9	6780	10 (321)	0.1	7571	22 (566)	0.3	8296	43 (936)	0.5	9064	86 (1691)	0.9	9818	55 (1735)	0.6
Veneto		62 (1777)		8711	105 (2686)	1.2	10,113	86 (2105)	0.9	11,327	97 (1546)	0.9	12,468	184 (4037)	1.5	14,155	128 (1978)	0.9	15,830	77 (1148)	0.5
**Total**		**104 (4664)**		**53,628**	**335 (7103)**	**0.6**	**65,678**	**272 (8106)**	**0.4**	**74,129**	**202 (4605)**	**0.3**	**82,245**	**268 (8456)**	**0.3**	**93,483**	**296 (5861)**	**0.3**	**101,854**	**327 (22,804)**	**0.3**

* Not available since the Italian national beekeeping registry was not yet operating. ** Extracted from the Italian national beekeeping registry.

## Data Availability

The data presented in this study are available on request from the corresponding author. The data are not publicly available due to privacy concerns.

## Supplementary Materials

The following supporting information can be downloaded at: https://www.mdpi.com/article/10.3390/insects13111059/s1, Table S1: Questions included in the study; Table S2: Coloss questionnaire; Table S3: Distribution of losses due to queen-related problems, natural disasters, and dead or empty colonies.
