# Supplementary material for: Some Considerations about Winter Colony Losses in Italy According to the Coloss Questionnaire
Source: Insects. 2022 Nov 16;13(11):1059. doi: 10.3390/insects13111059 (PMC9693309; doi:10.3390/insects13111059)
Supplement: Supplementary file 1 [file insects-13-01059-s001.zip › insects-1984543-supplementary/S1_Questions.pdf]

Supplementary Materials

Table S1: Questions included in the study.

Questions	Type of question and response options
1. To describe the location of your main apiary or operation, please state<br>a) the name of a city/town/village near to your apiary?<br>b) and the postal code of the apiary (or a postal code nearby)	Open question
2. How many production colonies did you have before winter *year1(y1)-year2(y2)*?	Open question (numerical entry)
In the next questions you are asked for numbers of colonies lost. Please consider a colony as lost if it is dead (or reduced to a few hundred bees), OR lost due to natural disaster, OR ALIVE but with queen problems, like drone laying queens or no queen at all, which you couldn't solve. EACH LOST COLONY SHOULD BE INCLUDED IN ONLY ONE OF THESE THREE CATEGORIES.	
3. How many of the colonies of question 2) still have live bees present but have unsolvable queen problems? If none, please answer 0	Open question (numerical entry)
4. How many of the colonies of question 2) did you lose because of natural disaster (suffocation from snow, flood, bear, theft…)? If none, please answer	Open question (numerical entry)
5. How many of the colonies of question 2) did you lose because they were dead or the hive was empty? If none, please answer 0	Open question (numerical entry)
6. Have you treated your colonies against Varroa during the period April *y1* - March *y2?*	Close-ended question (single choice): Yes; No; Don't know/not applicable
7. Could you please indicate the months when you monitored your production colonies for Varroa AND also indicate when you STARTED a Varroa treatment or management plan during the period April *y1* - March *y2*?<br>Treatments/management plan:<br>– Monitoring of Varroa infestation level (e.g. counting mite fall)<br>– Drone brood removal<br>– Hyperthermia (heat treatment of brood/bees)<br>– Other biotechnical method (as e.g. trapping comb, complete brood removal, queen confinement)<br>– Formic acid - short term<br>– Formic acid - long term (e.g. MAQS)<br>– Lactic acid	Close-ended question (multiple choice) for each treatment/management plan:<br>April *y1*; May *y1*; June *y1*; July *y1*; August *y1*; September *y1*; October *y1*; November *y1*; December *y1*; January *y2*; February *y2*; March *y2*

- Oxalic acid - trickling
- Oxalic acid - sublimation (evaporation)
- Oxalic acid mixtures (e.g. Hiveclean/Bienenwohl/Varromed)
- Thymol (e.g. Apiguard, ApilifeVar)
- Tau-fluvalinate (e.g. Apistan)
- Flumethrin (e.g. Bayvarol, Polyvar)
- Amitraz (in strips, e.g. Apivar, Apitraz)
- Amitraz (fumigation/aerosol)
- Coumaphos (e.g. Perizin)
- Coumaphos in strips(e.g. Checkmite+)
- Another chemical product
- Another method
