# Supplementary material for: Some Considerations about Winter Colony Losses in Italy According to the Coloss Questionnaire
Source: Insects. 2022 Nov 16;13(11):1059. doi: 10.3390/insects13111059 (PMC9693309; doi:10.3390/insects13111059)
Supplement: Supplementary file 1 [file insects-13-01059-s001.zip › insects-1984543-supplementary/S2_COLOSS_Questionnaire_2022.pdf]

Supplementary Material

Table S2 – Coloss questionnaire

Dear colleagues (national coordinators),

Below you will find the final version of the 2022 COLOSS questionnaire on honey bee colony losses.

If you want to monitor honey bee colony losses in your country, please use the questionnaire as suggested here. Note that some questions are of very high importance, hence please make sure to include those essential questions marked red (❶, ❹, ❺ etc). There are other questions which could also be included, and, if so, they should not be changed, because then we can compare the answers in the international analysis. These optional questions are marked with grey (⑩, ⑫, ⑮, 22 etc). Of course you can also add other questions that you might think can help in further analysing colony losses. Sentences marked with open circles (E①, E②, E③, E④ etc) are explanations and case definitions. Please also provide these to the beekeepers in a way that helps to maintain the high standards of our research.

If you are aiming at complete coverage of the beekeeper population rather than random sampling, we suggest using as many different ways to publicise, distribute and collect the survey questionnaire as possible to obtain the maximum sample size: Beekeeping journals, meetings, websites, social media etc. A simple and free of cost online tool (Limesurvey) has been made available this year for all countries who wish to use it, and you should contact the international coordinators for further information.

If you are monitoring colony losses in your country for the first time, we recommend that you contact the international coordinators so that a mentor can help you. You may wish to use only the essential questions until your survey is more established.

Important deadline: If you want your data to be considered for analysis and inclusion in the next international publication, you must include ALL essential questions marked with a red circle AND your data must be returned to Alison Gray (Data Coordinator) **and** Robert Brodschneider not later than **1st July 2022**. A reminder will be issued nearer the time. To standardise the datasets, standard coding of all your data in the so called "code book" is needed. This is a spreadsheet used for return of the data. You will receive the codebook in the next few weeks. If you do not receive this, or if you have any problems with re-coding, please contact us.

NOTE: To have your data included in any further data analysis, you must 1. Use all essential questions, 2. Check data for consistency (see below *****), 3. submit properly recoded data in our codebook (to be sent to you later) before the deadline.

International coordinators of the COLOSS core project on monitoring colony losses:
Robert Brodschneider [Robert.brodschneider@uni-graz.at]
Alison Gray [a.j.gray@strath.ac.uk]
Victoria Soroker [sorokerv@volcani.agri.gov.il]
Have you already signed up for our **Slack** workspace for communication?
https://colossmonitoring.slack.com

Put your own logo etc. here: remove these!

bienenstand.at

ZUKUNFT BIENE

Prevention of Honey Bee COlony LOSSes COLOSS

COLOSS honey bee colony loss and survival survey 2021/2022

www.COLOSS.org

Return or send this questionnaire to: EMAIL // FAX // ADDRESS // WEBSITE etc. before DEADLINE

* Consistency checks:

1. colonies at start of winter should be not missing and should be greater than zero
2. colonies lost should not be missing and should be greater than or equal to zero
3. dead colonies plus colonies lost due to queen problems plus colonies lost to natural disaster should not be greater than colonies at start of winter
4. Beekeepers with 1 apiary can't have their colonies more than 15 km away from each other, etc. Please also try to identify and remove any possible double entries.
5. Number of wintered colonies that had a new queen [in previous year] cannot be larger than number of colonies wintered!

Put your own logo etc. here:

bienenstand.at ZUKUNFT BIENE Prevention of Honey Bee Colony LOSSes COLOSS www.COLOSS.org

COLOSS honey bee colony loss and survival survey 2021/2022

1 To describe the location of your main apiary or operation, please state
  a) the name of a city/town/village near to your apiary? ____________
  b) and the postal code of the apiary (or a postal code nearby) ____________

2 How many apiaries do you have? [ ]

3 If you have more than one apiary, are all your apiaries within a distance of about 15 km of each other? If you have only one apiary, please answer yes.

O Yes O No O Don't know

E② Numbers of colonies, losses and status of the colony post-winter. Please consider winter as the period between the moment that you finished the pre-winter preparations for your colonies and the start of the new foraging season.
In this questionnaire we try to gather information about production colonies. Please consider colonies which are queenright and strong enough to provide a honey harvest as production colonies.

4 How many production colonies did you have before winter 2021-2022? [ ]

E③ In the next questions you are asked for numbers of colonies lost. Please consider a colony as lost if it is dead (or reduced to a few hundred bees), OR lost due to natural disaster, OR ALIVE but with queen problems, like drone laying queens or no queen at all, which you couldn't solve. EACH LOST COLONY SHOULD BE INCLUDED IN ONLY ONE OF THESE THREE CATEGORIES.

5 How many of these (4) colonies still have live bees present but have unsolvable queen problems. If none, please answer 0. [ ]

6 How many of these (4) colonies did you lose because of natural disaster (suffocation from snow, flood, bear, theft…)? If none, please answer 0. [ ]

7 How many of these (4) colonies did you lose because they were dead or the hive was empty? If none, please answer 0. [ ]

8 How many of the DEAD colonies or empty hives (7)
  a) … had many dead bees in or in front of the hive? [ ]
  b) … had no or only a few dead bees in or in front of the empty hive? [ ]
  c) … had dead workers in cells and no food present in the hive (signs of starvation)? [ ]
  d) … had dead workers in cells while food was present in the hive? [ ]
  e) … had none of the above or unknown symptoms? [ ]

PLEASE TREAT OPTIONS a) TO e) AS MUTUALLY EXCLUSIVE FOR ANY ONE COLONY.

9 How many of the wintered colonies (4) were weak but queenright after winter 2021-2022? [ ]

E④ We would like to calculate increases and decreases in the number of colonies, so if you had colonies in spring 2021 and remember how many you had, please answer the following two questions, considering spring as the start of the foraging season:

10 How many production colonies did you have in a) spring 2021 (last year)? [ ]
b) spring 2022? [ ]

COLOSS honey bee colony loss and survival survey 2021/2022

Return or send this questionnaire to: EMAIL // FAX // ADDRESS // WEBSITE etc. before DEADLINE

E⑤ Conditions in the colonies, the environment around the apiary, and management:

⓫ How many of the wintered colonies had a new queen in 2021? Don't know

⑫ To what extent did you observe queen problems in your colonies during the foraging season of 2021 compared to what you usually have?

O More O Normal O Less O Don't know

⓭ Did you migrate any of your colonies at least once for honey production or pollination in 2021?

O Yes O No O Don't know

⓮ Have you noticed bees with crippled/deformed wings in your colonies (during the summer season)? (These are signs of the presence of Deformed Wing Virus, which is spread by Varroa mites).

O Not at all O to a limited extent O to a large extent O Don't know

⑮ Did the majority of your bee colonies have a significant flow on one or more of the following sources in 2021?

a)	Orchards	O Yes	O No	O Don't know
b)	Oil seed rape	O Yes	O No	O Don't know
c)	Maize	O Yes	O No	O Don't know
d)	Sunflower	O Yes	O No	O Don't know
e)	Heather	O Yes	O No	O Don't know
f)	Autumn forage crops	O Yes	O No	O Don't know
g)	Honeydew	O Yes	O No	O Don't know
h)	Honeydew with melecitosis	O Yes	O No	O Don't know

⓰ Have you monitored your colonies for Varroa during the period April 2021 - March 2022?

O Yes O No O Don't know / not applicable

⓱ Have you treated your colonies against Varroa during the period April 2021 - March 2022?

O Yes O No O Don't know / not applicable

Put your own logo etc. here:

bienenstand.at ZUKUNFT BIENE Prevention of Honey Bee COlony LOSSes COLOSS www.COLOSS.org

COLOSS honey bee colony loss and survival survey 2021/2022

18 Could you please indicate the months when you monitored your production colonies for Varroa AND also indicate when you STARTED a *Varroa* treatment or management plan during the period April 2021 - March 2022?	Month in which each measure started											
	2021									2022		
**Method / Product**	April	May	June	July	August	September	October	November	December	January	February	March
Monitoring of Varroa infestation level (e.g. counting mite fall)												
Drone brood removal												
Hyperthermia (heat treatment of brood/bees)												
Other biotechnical method (as e.g. trapping comb, complete brood removal, queen confinement)												
Formic acid - short term												
Formic acid - long term (e.g. MAQS)												
Lactic acid												
Oxalic acid - trickling												
Oxalic acid - sublimation (evaporation)												
Oxalic acid mixtures (e.g. Hiveclean/Bienenwohl/Varromed)												
Thymol (e.g. Apiguard, ApilifeVar)												
Tau-fluvalinate (e.g. Apistan)												
Flumethrin (e.g. Bayvarol, Polyvar)												
Amitraz (in strips, e.g. Apivar, Apitraz)												
Amitraz (fumigation/aerosol)												
Coumaphos (e.g. Perizin)												
Coumaphos in strips(e.g. Checkmite+)												
Another chemical product												
Another method												

Put your own logo etc. here: remove these!

bienenstand.at

ZUKUNFT BIENE

www.COLOSS.org

COLOSS honey bee colony loss and survival survey 2021/2022

Return or send this questionnaire to: EMAIL // FAX // ADDRESS // WEBSITE etc. before DEADLINE

FURTHER OPTIONAL QUESTIONS (to be placed beside appropriate questions above)**:**

19 What is the name of the region where you keep your bees? ______________________

20 If you gave your colonies a supplemental sugar feed (sugar solution or inverted sugar) last year to prepare for winter, how many kg of sugar (dry matter) did you give on average per production colony? [ ]

21 Do you keep your bees in an area where Varroa has still not been detected?
O Yes O No O Don't know

22 What particular measures apply for the majority of your beekeeping:

a)	Screened bottom board in Winter	O Yes	O No	O Don't know
b)	Insulated hives in Winter (incl. double-walled hives)	O Yes	O No	O Don't know
c)	Hives made from synthetic materials	O Yes	O No	O Don't know
d)	Certified organic beekeeping	O Yes	O No	O Don't know
e)	Queens bred from Varroa-tolerant/resistant stock	O Yes	O No	O Don't know
f)	Small brood cell size (5.1 mm or less)	O Yes	O No	O Don't know
g)	Comb without foundation	O Yes	O No	O Don't know
h)	Purchase wax from outside own operation	O Yes	O No	O Don't know
i)	Non-wax foundation in brood chamber	O Yes	O No	O Don't know

23 Did you observe *Vespa velutina* foraging for honey bees in your apiary/apiaries?
O Yes O No O Don't know

24 How much (in local currency) do you estimate your costs to be per colony for Varroa treatment in the period April 2021 – March 2022?

25 What methods did you use to monitor your production colonies for Varroa during the 2021/22 season? (Check all that apply)

O Natural mite fall on bottom board
O Visual inspection of adult bees
O De-capping brood
O Adult bees: Sugar shake / roll
O Adult bees: washing with detergent
O Adult bees: CO_2
O Sent sample to lab
O Other (enter explanation): ______________________

26 Please identify in which months… (choose any that apply)

	Apr	May	Jun	Jul	Aug	Sep	Oct	Nov	Dec	Jan	Feb	Mar	Not at all
… you add supers													
… you change queens													
… you split colonies													
… you merge colonies													
… you harvest honey													
… you often experience colony losses													
… you manage the highest number of colonies													
… you manage the lowest number of colonies													
… colonies swarm													
… colonies abscond													

… colonies rely on food stores only													

Do not forget to thank your beekeepers very much!
