# Supplementary material for: Some Considerations about Winter Colony Losses in Italy According to the Coloss Questionnaire
Source: Insects. 2022 Nov 16;13(11):1059. doi: 10.3390/insects13111059 (PMC9693309; doi:10.3390/insects13111059)
Supplement: Supplementary file 1 [file insects-13-01059-s001.zip › insects-1984543-supplementary/S3_Distribution of losses due to queen-related problems, natural disaster, and dead or empty colonies..pdf]

Supplementary Materials

Table S3: Distribution of losses due to queen-related problems, natural disaster, and dead or empty colonies.

Region	2014-2015					2015-2016					2016-2017					2017-2018					2018-2019					2019-2020					2020-2021				
	N. colonies	Lostqproblem	Lostnatdisaster	LostDeadEmpty	N. Lost colonies (%)	N. colonies	Lostqproblem	Lostnatdisaster	LostDeadEmpty	N. Lost colonies (%)	N. colonies	Lostqproblem	Lostnatdisaster	LostDeadEmpty	N. Lost colonies (%)	N. colonies	Lostqproblem	Lostnatdisaster	LostDeadEmpty	N. Lost colonies (%)	N. colonies	Lostqproblem	Lostnatdisaster	LostDeadEmpty	N. Lost colonies (%)	N. colonies	Lostqproblem	Lostnatdisaster	LostDeadEmpty	N. Lost colonies (%)	N. colonies	Lostqproblem	Lostnatdisaster	LostDeadEmpty	N. Lost colonies (%)
Emilia Romagna	27	0	0	0	0 (0.0%)	145	5	3	17	25 (17.2%)	1063	23	0	90	113 (10.6%)	646	54	2	211	267 (41.3%)	162	8	0	11	19 (11.7%)	688	36	12	47	95 (13.8%)	2971	135	0	213	348 (11.7%)
Friuli Venezia Giulia	2443	143	18	335	496 (20.3%	36	4	2	3	9 (25.0%)	1134	0	0	153	153 (13.5%)	22	1	0	6	7 (31.8%)	104	17	0	21	38 (36.5%)	400	26	1	56	83 (20.8%)	1099	67	46	180	293 (26.7%)
Lombardia	128	1	0	14	15 (11.7%)	2725	170	33	204	407 (14.9%)	1867	155	8	306	469 (25.1%)	1046	56	0	113	169 (16.2%)	156	1	0	11	12 (7.7%)	166	5	0	29	34 (20.5%)	7957	368	247	875	1490 (18.7%)
Piemonte	10	0		0	0 (0.0%)	50	1		4	5 (10.0%)	193	1	0	26	27 (14.0%)	664	77	21	79	177 (26.7%)	479	7	0	27	34 (7.1%)	648	34	0	145	179 (27.6%)	1567	164	5	159	328 (20.9%)
Sicilia	125	2	9	9	20 (16.0%)	195	5	5	9	19 (9.7%)	1423	110	50	33	193 (13.6%)	115	10	0	22	32 (27.8%)	2582	24	84	104	212 (8.2%)	290	12	0	38	50 (17.2%)	6327	147	84	532	763 (12.1%)
Trentino Alto Adige	154	4	0	13	17 (11.0%)	1266	62	22	76	160 (12.6%)	321	58	0	91	149 (46.4%)	566	32	0	86	118 (20.8%)	936	54	5	114	173 (18.5%)	1691	168	10	164	342 (20.2%)	1735	137	18	243	398 (22.9%)
Veneto	1777	85	21	441	547 (30.8%)	2686	160	45	163	368 (13.7%)	2105	169	24	295	488 (23.2%)	1546	126	6	221	353 (22.8%)	4037	201	38	474	713 (17.7%)	1978	152	8	202	362 (18.3%)	1148	112	16	201	329 (28.7%)
**Total**	**4664**	**235**	**48**	**812**	**1095 (23.5%)**	**7103**	**407**	**110**	**476**	**993 (14.0%)**	**8106**	**516**	**82**	**994**	**1592 (19.6%)**	**4605**	**356**	**29**	**738**	**1123 (24.4%**	**8456**	**312**	**127**	**762**	**1201 (14.2%)**	**5861**	**433**	**31**	**681**	**1145 (19.5%)**	**22804**	**1130**	**416**	**2403**	**3949 (17.3%)**
